# On Diseases of Menstruation and Ovarian Inflammation in Connexion with Sterility, Pelvic Tumors, and Affections of the Womb

**Published:** 1851-07

**Authors:** 


ARTICLE IV.

*On Diseases of Menstruation and Ovarian Inflammation in connexion with Sterility, Pelvic Tumors, and Affections of the Womb:*—By EDWARD JOHN TILT, M.D., Physician to the Farmington General Dispensary, and to the Paddington free Dispensary for the Diseases of Women and Children, "Omne Animal ab Ovo." pp 285: 12 mo: New-York. S. S. & W. Wood, No. 261 Pearl St.: 1851: (from the publishers.)

We have been looking with some anxiety for the appearance of this work, materials for which were being gathered by the author, by a circular of enquiry, about a year ago. From a glance at its design and general arrangement, we are very favorably impressed, and have no doubt it will prove to be a valuable work. As it is something new, we will endeavor to give an anlysis of it in our next number; in the mean time we advise our readers who wish to post up on the topics indicated in the title at the head of this article, to procure the work and examine it for themselves.

